# The Impact of Harvest Season on Oolong Tea Aroma Profile and Quality

**DOI:** 10.3390/plants14152378

**Published:** 2025-08-01

**Authors:** Chao Zheng, Shuilian Gao, Xiaxia Wang, Zhenbiao Yang, Junling Zhou, Ying Liu

**Affiliations:** 1 Haixia Institute of Science and Technology, Fujian Agriculture and Forestry University, Fuzhou 350007, China; zhengchaotea@fafu.edu.cn (C.Z.);; 2 Anxi College of Tea Science, Fujian Agriculture and Forestry University, Fuzhou 350007, China

**Keywords:** oolong tea, aroma quality, season, HS-SPME-GC-MS, untargeted metabolomics

## Abstract

The impact of seasonality on the aroma quality of tea has been documented in various tea types, but not specifically in oolong tea. This study is the first to explore the complex relationships between seasonality, volatile compounds, and aroma quality in oolong tea. Using Headspace Solid-Phase Microextraction Gas Chromatography–Mass Spectrometry (HS-SPME-GC-MS)-based untargeted metabolomics, we analyzed 266 samples of Tieguanyin oolong tea. The data identified linalool, linalool oxides (trans-linalool oxide (furanoid) and trans-linalool oxide (pyranoid)), and their metabolites (diendiol I; hotrienol) as key seasonal discriminants. Four out of the top ten key differential compounds for distinguishing aroma scores were metabolites from fatty acid degradation, namely trans-3-hexenyl butyrate, trans-2-hexenyl hexanoate, hexyl hexanoate, and hexyl 2-methyl butyrate. Approximately one-fifth of the seasonal discriminant volatile compounds were significant in influencing aroma quality. Overall, the impact of seasonality on the aroma quality of finished Tieguanyin oolong tea is marginal. These findings enhance our understanding of the interplay between seasonal variations, volatile composition, and aroma quality in oolong tea.

## 1. Introduction

Tea is universally consumed and appreciated for its health, sensory, relaxing, and cultural characteristics. This subtropical plant, *Camellia sinensis*, is susceptible to climate challenges, which subsequently impact the quality of its finished products. For example, for oolong tea, even though summer and autumn leaves contribute to over 60% of the total annual tea output, they are generally sold at lower prices, particularly the summer tea, due to inferior quality. Consumers reported different sensory perceptions towards tea made from different seasons. For instance, Tieguanyin, a widely consumed oolong variety in China, exhibits a more intense and pleasant aroma in its autumn type compared to the spring tea [1].

Our previous research has investigated oolong tea’s seasonal variations and sensory profiles based on untargeted metabolomics on non-volatile compounds [2]. We discovered that half of the identified key non-volatile compounds are also crucial for determining the sensory grade, indicating the significant impact of seasonality on sensory quality [2]. Triterpene saponins synthesized from the mevalonate pathway were recognized for the first time as critical factors in determining both the harvest season and sensory grade of oolong tea [2]. Now, the question remains whether such a seasonal impact will lead to changes in aroma profile and quality.

The differences in aroma profiles and volatile compound compositions among various tea types, such as green tea [3], white tea [4], and black tea [5,6], have been widely studied. Research shows that seasonal variations in volatile organic compounds (VOCs) present distinct differences across these tea types. However, there is currently no research examining how seasonality affects the volatile metabolite profiles of oolong tea, or how these changes impact aroma perception. Given that aroma is a crucial factor in the evaluation of tea, it is economically important to address this question. Furthermore, understanding the seasonal effects is essential for developing strategies to maintain high-quality tea in the context of climate change.

This study builds on our previous work to investigate how volatile compounds influence the aroma quality of oolong tea (Tieguanyin). We conducted untargeted GC-MS analyses on 266 finished tea products and paired these data with comprehensive aroma evaluations. This work addresses a critical gap in tea science: while seasonal variations are known to affect tea growth and preliminary chemistry, their impact on the final aroma profile of processed oolong tea remains poorly characterized. By employing a large, representative sample set, we enhance the statistical robustness of our findings, offering novel insights into how the harvest season modulates the sensory quality of finished tea products.

## 2. Results and Discussion

### 2.1. Seasonal Variation in Volatile Profiles of Tieguanyin Oolong Tea

Oolong tea products (*Camellia sinensis* var. *sinesis* cv. ‘Tieguanyin’) made from leaves collected in the autumn of 2020 and the spring of 2021 were obtained for a comprehensive study to understand the influence of seasonal variation on the volatile profiles of tea. HS-SPME-GC-MS was utilized to study 266 tea samples (three replicates each) and the GC/MS revealed significant differences between the two harvest seasons. A total of 181 volatile compounds were identified by comparing their mass spectra and retention indices with those in the NIST 2017 database, a house database, and standard reference materials.

While no compounds were specific to a single season, their abundance levels varied significantly between the two groups, with alcohol and terpenoids making up over 80% of the total volatile compounds. Since the amount of analyte extracted via SPME depends on many factors such as fiber coating affinity, sample matrix effects, and competition between analytes, it does not fully recover analytes, leading to the relative rather than absolute quantification of the compound; therefore, comparison between compound concentrations should be taken with caution. With that in mind, the most abundant volatile organic compounds (VOCs) identified in this study were “α-farnesene”, “nerolidol”, and “indole”, which align with other studies on Tieguanyin aroma profiles [7].

**Figure 1:**
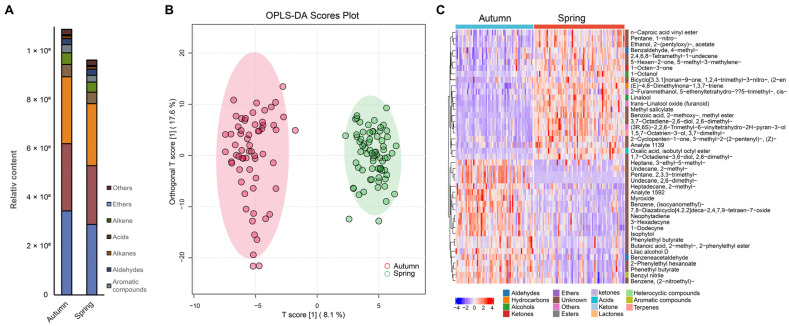
The aromatic profiles (**A**) of different seasons of light-scented Tieguanyin tea; OPLS-DA score plot (**B**) of finished light-scented Tieguanyin tea made from spring-harvested (*n* = 73 × 3) and autumn harvested (*n* = 60 × 3) leaves; heat map (**C**) of normalized accumulation levels of 64 differential metabolites between spring- and autumn-harvested light-scented Tieguanyin using OPLS-DA with VIP > 1.5, *p* < 0.05.

Other major VOCs included “(Z)-6-(pent-2-en-1-yl) tetrahydro-2H-pyran-2-one”, “2-phenylethanol”, “6-methylhept-5-en-2-one”, and “2-nitroethylbenzene”. These compounds have also been reported in other studies, although they were not reported as the top abundant VOCs in Tieguanyin [7,8]. We observed a great diversity among the Tieguanyin VOCs even within the same group (Figure 1 and Figure S1). Many factors could contribute to this diversity, such as the micro-environmental differences among tea plants and variations in the manufacturing process. In this study, we can better capture this diversity and generate more representative profiles of Tieguanyin tea, since 266 samples were analyzed in our research, compared to up to 25 samples in previous studies.

Due to the distinct differences in aroma between strong-scented and light-scented oolong tea, the seasonal comparison was carried out within each group instead of across the entire sample. Initially, the data underwent Principal Component Analysis (PCA) to determine if the volatile metabolites could be separated by season. The PCA plots indicated that the tea products made from leaves harvested in autumn and spring showed considerable overlap, suggesting a high similarity in the volatile profiles between the two seasons (Supplementary Figure S1).

In the Orthogonal Partial Least Squares Discriminant Analysis (OPLS-DA) models, we successfully separated the two seasons in the light-scented tea (Figure 1), but not in the strong-scented tea. The OPLS-DA models were validated using 300 permutation tests to assess the risk of overfitting. For the light-scented tea model, the permutation test yielded R^2^Y = 0.954 and Q^2^ = 0.908 (Supplementary Figure S2), confirming the model’s validity and absence of overfitting. OPLS-DA failed to discriminate strong-scented tea samples, likely due to the high-temperature processing (roasting/baking), reducing volatile compound diversity and creating more homogeneous profiles between seasons. Consequently, the study primarily focused on light-scented tea. This observation aligns with our non-volatile metabolomic studies, which revealed reduced seasonality in strong-scented tea [2]. The high-temperature baking and roasting processes during manufacturing inevitably lead to a significant loss or transformation of volatile compounds.

We identified 64 metabolites that significantly contributed to the seasonal variation in volatile profiles for light-scented tea, based on the univariate significance threshold (*p* < 0.05) and the multivariate VIP (Variable Importance in Projection) > 1 rule (Figure 1C, Supplementary Table S2). The top 10 metabolites with the most significant changes were “trans-linalool oxide (furanoid) (linalool oxide II)”, “methyl salicylate”, “trans-linalool oxide (pyranoid) (linalool oxide IV)”, “benzoic acid, 2-methoxy-, methyl ester”, “diendiol I”, “hotrienol”, “ethanol, 2-(pentyloxy)-, acetate”, “linalool”, “isophytol”, and “2-phenylethyl hexanoate”.

The seasonal variation in volatile organic compounds (VOCs) demonstrates distinct differences across different tea types. For instance, in black tea, key seasonal differential aroma compounds include linalool, ocimene, benzeneacetaldehyde, and methyl salicylate, along with 37 other identified compounds. For white tea harvested in spring and autumn, the principal differential VOCs comprise 2-hexenal, 1-hepten-6-one, 2-methyl-, (E)-4,8-dimethylnona-1,3,7-triene, and 51 additional compounds [4]. Our study provides the first list of volatile metabolites for differentiating harvesting seasons in oolong tea.

Half of these key compounds belonged to linalool-related metabolites (namely “trans-linalool oxide (furanoid)”, “trans-linalool oxide (pyranoid)”, “diendiol I”, “hotrienol”, and “linalool”), known for their floral sensory attributes. These compounds were more abundant in the spring products, contributing to the “tea-like” aroma characteristic of spring tea (Figure 1C). Even though they are the most reported linalool-derived aroma compounds in tea regardless of type or variety [9], the levels of linalool and its related compounds were much lower in oolong tea than in black tea. This could be attributed to glycoside hydrolysis during the rolling and fermentation processes, which is characteristic of oolong tea as a semi-fermented tea product. Zeng et al. [7] found that linalool is a key differentiating volatile of Tieguanyin in terms of determining the type of tea, with Tieguanyin having lower levels of linalool. Despite lower linalool levels in Tieguanyin, it remains a strong odor component in oolong tea [10].

Linalool synthesis happens in the chloroplasts of tea plants through the methylerythritol phosphate (MEP) pathway, with geranyl pyrophosphate (GPP) as the key precursor and CsLIS responsible for the transformation of GPP into linalool [9] (Supplementary Figure S3). Linalool’s transformation into linalool oxides is a light-dependent process, with more light resulting in less transformation [9]. Diendiol I is a diol derivative of linalool after linalool undergoes hydroxylation. The formation of diendiol I has been reported to be related to tea green leafhopper *Empoasca onukii* attack [9]. The level of diendiol I increased linearly with increasing leafhopper density [11]. The occurrence of this leafhopper is observed when the temperature is about 25 °C and the environment is humid. The climatic variation between spring and autumn will likely impact the insect activity, thereby influencing the diendiol I content [12]. Hotrienol is a tertiary alcohol formed through the oxidation and enzymatic transformation of linalool. Several studies indicated that hotrienol levels were strongly associated with altitude [13,14], but all the samples were made from tea plants grown in similar altitudes in the study. The impact of seasonality on the level of hotrienol in tea has not been studied or reported. Studies showed that linalool and its oxides, as well as diendiol I, do not change significantly during the oolong tea manufacturing process [10].

Seasonal changes reflect temperature, light intensity, and precipitation differences. The average spring harvesting season for Tieguanyin oolong ranges from mid-April to mid-May, and the autumn season is from mid-September to mid-October. Based on the local weather of Anxi in 2020 and 2021, the spring season has temperatures between 19.57 °C and 29.57 °C, precipitation of 409 mm, and an average day length of 13 h, while the autumn season has temperatures between 20.43 °C and 29.33 °C, precipitation of 4.19 mm, and a day length of 12 h (https://tianqi.2345.com/wea_history/70016.htm, accessed on 2 May 2025). While the temperature variation between the two seasons is minimal and the difference in day length is small, the perception between the two seasons differed substantially, with autumn exhibiting significantly drier conditions than spring. For instance, the leafhopper density will likely be higher in spring as a result of humid conditions, which might have led to the higher diendiol I levels observed in the study. Gouinguene and Turlings [15] showed that the number of volatiles released by corn plants decreases as the humidity of the air or soil increases. High humidity and warm weather conditions favor faster growth with lower dry matter accumulation per shoot volume [16]. These agreed with our finding that spring tea (higher humidity) has a lower amount of VOCs than autumn tea.

### 2.2. Machine Learning Algorithms for Distinguishing Seasons

The application of machine learning has been widely used in tea research to distinguish properties like variety, geographical origin, and processing [17] for authenticity and quality control purposes [18,19]. As previously mentioned, tea products from different seasons are often sold at varying prices; thus, a quality control system would be incomplete without the ability to distinguish seasonal differences. Here, we developed a discrimination model based on the gradient boosting (GB) algorithm to differentiate seasonal variations in light-scented Tieguanyin oolong tea. Specifically, we used 133 light-scented tea samples with all 181 identified volatile features as input variables for model construction. As an ensemble learning method, gradient boosting iteratively combines weak learners to create a strong predictive model by minimizing loss functions in a gradient descent manner, making it particularly effective for classification tasks [20,21]. While univariate analysis (OPLS-DA) identified significant VOC differences between seasons, machine learning provides several additional benefits: (1) it captures complex non-linear relationships and interactions between multiple VOCs simultaneously, rather than examining compounds in isolation; (2) it provides a predictive capability for classifying unknown samples; and (3) it offers feature importance ranking through SHAP analysis, revealing which compounds contribute most to classification decisions. Considering the possibility of overfitting based on sample size, we implemented several overfitting prevention measures such as 5-fold cross-validation during training, hyperparameter optimization through GridSearchCV, and external validation on a held-out test set.

**Figure 2:**
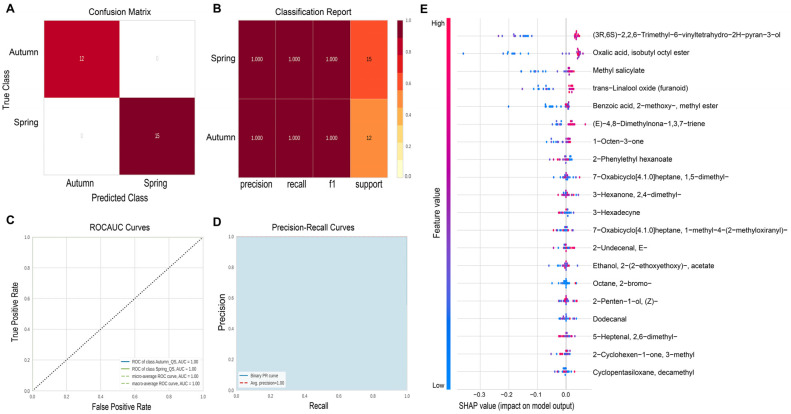
Machine learning model performance for seasonal classification of light-scented Tieguanyin tea using 133 samples and 181 volatile features. The confusion matrix (**A**) and the classification report (**B**) of the validation set samples; the receiver operating characteristic (ROC) curve (**C**) and the precision–recall (PR) curves (**D**) of GB model; Shapley additive explanations (SHAP) plot (**E**) for the gradient boosting model. The bar graph shows the input variables’ relative importance. The figure plots every sample in the analysis as a point. The y-axis lists the input variables. The x-axis is a metric of the SHAP value associated with each variable and sample within the dataset (i.e., points plotted for each case based on the impact on prediction). The points plotted on the far left have a greater impact on X prediction, and points plotted on the right have a greater impact on Y prediction. The normalized value of observation is color-based (red = higher values; blue = lower values).

The dataset of 133 light-scented tea samples (with 181 volatile features) was randomly split into two parts in a stratified fashion: 80% (106 samples) for model training and 20% (27 samples) for model validation. The model yielded a prediction accuracy of 99.1% based on five-fold cross-validation (Figure 2A,B). Given the promising performance of GB on internal cross-validation, a test set was built with the remaining 20% of samples (27 in total) for external validation. All 15 autumn-harvested tea samples and 12 spring-harvested tea samples from the test set were correctly classified. Overall, these results demonstrate the effectiveness of VOC metabolomics in tandem with ML-based algorithms for analyzing seasonal variation in Tieguanyin oolong tea.

To assess the individual contribution of each feature to the model’s performance, we employed SHAP (SHapley Additive exPlanations) values as a feature ranking tool. SHAP provides an interpretable framework that quantifies the unique and additive impact of each feature on the final classification outcome (Figure 2E). The top five most influential features identified via SHAP analysis were trans-linalool oxide (pyranoid), oxalic acid isobutyl octyl ester, methyl salicylate, trans-linalool oxide (furanoid), and benzoic acid 2-methoxy-methyl ester. Four of the top five compounds (i.e., trans-linalool oxide (pyranoid), methyl salicylate, trans-linalool oxide (furanoid), and benzoic acid 2-methoxy-methyl ester) were also identified among the top five most significant features via OPLS-DA, demonstrating consistency between the two analytical approaches, and reconfirming their importance in distinguishing seasonality.

### 2.3. Relationship Between Volatile Metabolites and Sensory Quality

**Figure 3:**
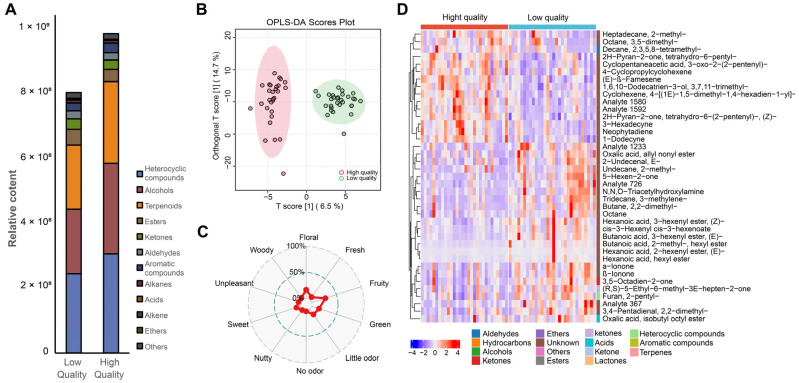
The aromatic profiles (**A**), OPLS-DA score plot (**B**), and odors related to the key differentiating metabolites (**C**) of high-quality (top 30 in aroma score) and low-quality (bottom 30 in aroma score) grades of Tieguanyin tea; heat map (**D**) of normalized accumulation levels of 33 differential metabolites between high- and low-quality Tieguanyin tea using OPLS-DA with VIP > 1, *p <* 0.05.

To investigate the relationship between seasonal variations in metabolites and tea quality, we conducted comprehensive sensory evaluations of 266 Tieguanyin tea products. We evaluated light-scented and strong-scented tea separately due to their distinct characteristics. For the strong-scented teas, we found no significant separation of metabolites between high- and low-quality samples. However, a clear distinction emerged between high-quality and low-quality light-scented teas using OPLS-DA models. This is consistent with the non-volatile metabolomic data, where light-scented tea showed more distinct separation between higher and lower grades. Our observations indicated that higher-quality teas had a higher ratio of terpenoids, heterocyclic compounds, and alcohols, while lower-quality Tieguanyin oolong teas contained elevated levels of acids, esters, ketones, aldehydes, and alkanes (Figure 3A).

Using OPLS-DA (with criteria of VIP > 1 and *p* < 0.05), a total of 31 metabolites were identified as key differentiating metabolites (Figure 3B,D, Supplementary Table S3). Terpenoids and esters made up half of the key differentiating metabolites, with common odors related to fresh, green, and fruity notes (Figure 3C). The top ten known metabolites were trans-3-hexenyl butyrate; (E)-β-Farnesene; trans-2-hexenyl hexanoate; hexyl hexanoate; hexyl 2-methyl butyrate; nerolidol; cis-α-bisabolene; and neophytadiene.

Approximately half of these compounds (trans-3-hexenyl butyrate, trans-2-hexenyl hexanoate, hexyl hexanoate, and hexyl 2-methyl butyrate) were esters derived from fatty acid degradation pathways, which are known for providing fresh, green, and fruity aromas [22]. Jin et al., 2023 provided an excellent scheme of the pathway and explained in great detail how these degradation products serve as general response signals to various stress factors, including low temperature, drought, and pest attacks [22]. The odor threshold of these specific compounds has not been established; however, they are generally considered to have low odor thresholds [23]. Furthermore, they also show synergistic effects related to aroma contributions [24]. For example, when trans-2-hexenyl hexanoate was prepared below the odor threshold, it had no aroma. However, when just 5% of this compound was replaced with 4-hexanolide (another aroma-active compound found in oolong tea), the odor intensity was significantly enhanced [25]. Together, our data further supports the importance of these compounds in determining aroma quality.

Previous research on Tieguanyin tea has identified several potent odorants (flavor dilution factor ≥ 44), including trans-3-hexenyl butyrate, trans-nerolidol, indole, and jasmine lactone [26]. Zeng et al. (2022) demonstrated a positive correlation between trans-nerolidol concentrations and commercial tea grades. In their subsequent metabolomics and sensory evaluation study, Zeng et al. (2023) identified benzyl benzoate, β-phenylethyl acetate, 6-methyl-5-hepten-2-one, heptanal, and (E)-2-hexenal as potential grade discriminators [7,8]. While some volatile organic compounds (VOCs) identified in our study aligned with their findings (α-lonone, β-lonone, 3,5-octadien-2-one, and 3,5-octadien-2-one, (E, E)-), the key grade-discriminating VOCs largely differed. This discrepancy can be attributed primarily to the substantial difference in sample sizes. Given the considerable diversity observed in Tieguanyin samples, the expansion of sample sizes from less than 10 in their studies to over 100 in our investigation likely accounts for these divergent conclusions.

### 2.4. Combined Aroma, Seasonality, and Sensory Data

**Figure 4:**
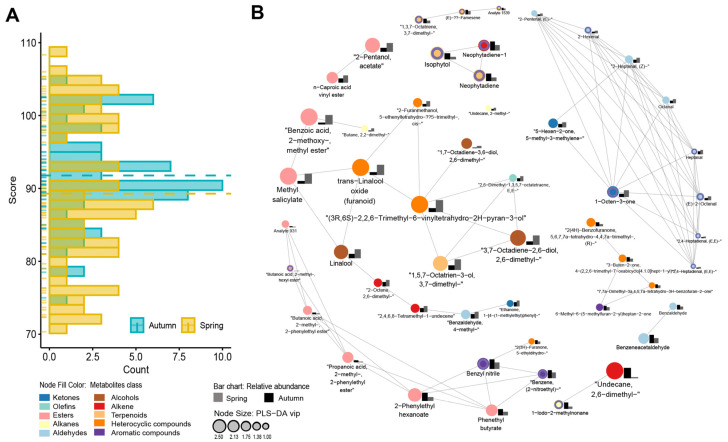
Histogram of the sensory scores (**A**) for the light-scented Tieguanyin tea collected from different seasons. The dotted line represents the average score of the season; correlation network of sensory-related metabolites (**B**) for light-scented Tieguanyin (|r| > 0.7 and *p* < 0.05) on VIP > 1 metabolites identified in OPLS-DA analysis. The purple ring indicates the compounds are important in both sensory and seasonality differentiation.

The integration of aroma profiles, seasonal patterns, and sensory evaluation data indicated that spring tea samples had slightly lower aroma scores compared to those harvested in autumn. Autumn tea products showed higher mean concentrations of volatile organic compounds (VOCs), which likely enhanced their more intense aromatic profiles. These findings support the common belief that autumn Tieguanyin tea is superior in aroma, while spring harvests are recognized for their exceptional taste characteristics (Figure 4). Out of the 33 differential compounds that distinguished higher- and lower-grade Tieguanyin tea, 9 compounds overlapped with the seasonal differential compounds. Six of these compounds, namely “(E)-β-farnesene, neophytadiene”, “(E)-2-undecenal”, “cis-α-bisabolene”, “isophytol”, “1-dodecyne”, and “hexyl 2-methylbutyrate” were higher in the autumn products. The remaining three compounds hexyl 2-methylbutyrate; 2-methylundecane; and oxalic acid, allyl nonyl ester were more abundant in the spring products. Two-way ANOVA revealed that while both season (F(1,56) = 27.95, *p* < 0.001, partial η^2^ = 0.33) and grade (F(1,56) = 867.27, *p* < 0.001, partial η^2^ = 0.94) significantly affected aroma scores, grade was the dominant factor, explaining 94% of the variance. The season × grade interaction was non-significant (F(1,56) = 1.76, *p* = 0.19, partial η^2^ = 0.03). Although autumn samples showed slightly higher mean aroma scores than spring samples, this seasonal effect (33% variance explained) was marginal compared to the overwhelming influence of tea grade.

Among these six compounds, (E)-β-famesene has been repeatedly mentioned as important in sensory quality studies across tea types. In oolong tea research, (E)-2-undecenal, (E)-β-famesene, indole, hexanoic acid hexyl ester, trans-2-hexenyl hexanoate, α-ionone, and β-ionone have demonstrated strong correlations with electronic nose array responses and significantly influenced sensory scores [27]. In black tea, (E)-β-farnesene and β-ionone were found to be significant contributors to perceived aroma quality [28]. In green tea, Lin et al. (2012) reported that (E)-β-farnesene exhibited strong positive correlations with aroma scores, while β-ionone showed strong negative correlations in the Longjing variety [29]. Considering its dual role in determining seasonality, this compound may serve as a marker for monitoring seasonal effects on aroma quality.

In conclusion, this study provides the first comprehensive investigation into the impact of harvest season on the aroma quality and volatile organic compound (VOC) profile of oolong tea (cultivar: Tieguanyin). By utilizing untargeted metabolomics and chemometric modeling, we identified linalool and its related metabolites as key compounds differentiating between harvest seasons. The levels of these metabolites are much higher in the tea made from spring-harvested leaves. Additionally, the comparison between high- and low-quality oolong tea revealed that the top key quality-differentiating compounds are derived from fatty acid degradation pathways. Optimizing the manufacturing of oolong tea, such as extending withering, oxidation, and tossing processes, may help release lipases and break down fatty acids. Our findings observed a marginal impact of seasonality on aroma scores. The insights gained from this study enhance our understanding of how seasonal changes impact tea aroma development. The influence of seasonality can directly affect the biosynthesis of VOCs due to climatic factors like temperature and precipitation. Additionally, it can impact insect activity, which increases the release of wounding compounds. These observations are vital for developing strategies to control the production of desired compounds and improve tea production practices.

## 3. Materials and Methods

### 3.1. Reagents and Materials

The 2-acetylpyrrole and n-alkane standard solutions C8-C25 were procured from Sigma-Aldrich (St. Louis, MO, USA). Additionally, 2-acetyl-5-methylfuran, β-phenylethyl butyrate, 2-phenylethyl hexanoate, ethyl isopropyl ketone, α-terpineol, and trans-2-hexenyl caproate were obtained from Shanghai Yuanye Bio-Technology Co., Ltd. (Shanghai, China). Hexyl hexanoate was sourced from Shanghai Zhenzhun Bio-Technology Co., Ltd. (Shanghai, China), while 4-methyl-3-penten-2-one came from Shanghai Yi’en Chemical Technology Co., Ltd. (Shanghai, China). Finally, ethyl decanoate was acquired from Shanghai ANPEL Scientific Instrument Co., Ltd. (Shanghai, China).

### 3.2. Sample Collection

A total of 266 oolong tea products (cultivar: Anxi Tieguanyin, types: light-scented (Qingxiang) and strong-scented (Nongxiang)) were gathered from 33 distinct tea production sites/companies in Anxi City, Fujian Province, China. The collection took place during the autumn of 2020 and the spring of 2021. Detailed sample collection information was provided in Supplementary Table S1. Samples were systematically gathered across various price ranges. After collecting, all tea samples were vacuum-sealed and stored in a refrigerator at −18 °C (0 °F).

### 3.3. Untargeted Volatile Metabolite Analysis

The volatile compounds in the tea samples were analyzed via HS-SPME-GC-MS (Headspace Solid-Phase Microextraction Gas Chromatography–Mass Spectrometry) following a previously established method in the lab [30,31].

Briefly, 2 g of tea sample was finely ground and mixed with 1 µg of internal standard (ethyl decanoate). The samples were incubated at 80 °C for 30 min, followed by another 60 min extraction and the collection of 5 volatile compounds at 80 °C with 1 cm, 65 µm polydimethylsiloxane-divinylbenzene (PDMS-DVB) solid-phase microextraction (SPME) fiber (Supelco, Bellefonte, PA, USA) before GC-MS analysis. An Agilent 7890B Gas Chromatograph (Agilent Co., Santa Clara, CA, USA) coupled with a Pegasus HT time-of-flight mass spectrometer (LECO Co., Saint Joseph, MI, USA) (GC-TOF MS) was used to determine the volatile profile. GC separation was achieved on a Restek Rxi^®^-5Sil MS capillary column (Shanghai, China) (30 m, inner diameter 0.25 mm and film thickness 0.25 µm). The oven temperature was programmed at 50 °C for 5 min, increased at 3 °C/min to 210 °C, then increased at 15 °C/min to 330 °C, and kept for a 5 min final hold. The MS was operated in an electron impact (EI) mode with an ionization energy of 70 eV and a mass scan range of 30–500 *m*/*z*. QC (quality control) samples prepared by pooling aliquots of all samples were injected every ten runs throughout the analysis to monitor instrument fluctuations.

The raw data underwent processing using ChromaTOF software (version 4.51.6, LECO) for spectral deconvolution and alignment. The following criteria were used: (1) signal-to-noise (S/N) ratio = 10; (2) maximum retention time difference = 2 s; (3) peak width = 5 s; and (4) mass spectral match score ≥ 650. Relative quantification was determined based on the peak area ratio of analytes to the internal standard.

### 3.4. Aroma Sensory Evaluation

The aroma sensory quality of the tea samples was evaluated by a team of certified tea evaluators from the Tea Scientific Society of China. The aroma scores were determined by the certified evaluators based on the fragrance intensity, lingering duration, freshness, and purity of the drink. The evaluation followed the Gaiwan method, in accordance with the Chinese standard GB/T 30357.2-2013 [32]. Specifically, 5g of Tieguanyin tea was brewed in a covered bowl (Gaiwan) with 110 mL of water. The fragrance of the tea was assessed and scored over three consecutive brews. All samples were evaluated on the same day, with ten samples in one group. Two reference sample sets were used in the evaluation: one was certified tea materials for each grade provided by Anxi Tieguanyin Association and the other one was the highest scored sample from the previous group.

Ethical approval was not required for this study as participants provided verbal consent for their participation and the use of their information. Participants were informed of their right to withdraw from the study at any time and were given a comprehensive disclosure of the study requirements and any potential risks.

### 3.5. Statistical Analysis

Data (266 tieguanyin tea samples) were QC-normalized, log10-transformed, and auto-scaled (mean-centered and divided by the standard deviation of each variable) before PCA (Principal Component Analysis) and OPLS-DA (Orthogonal Partial Least Squares Discriminant Analysis) in MetaboAnalyst (v5.0). Metabolites were considered significantly different between groups based on the following criteria: (1) Variable Importance in Projection (VIP) score > 1 from the OPLS-DA model, indicating substantial contribution to group separation; and (2) Benjamini–Hochberg false discovery rate (FDR)-adjusted *p*-value < 0.05 from univariate analysis, controlling for multiple testing. This combination of multivariate (VIP) and univariate (FDR-adjusted *p*-value) criteria ensures the robust identification of differential metabolites while minimizing false positives, following established metabolomics best practices (Worley & Powers, 2013, Current Metabolomics) [33]. Heatmaps were generated using the R package ComplexHeatmap (v2.16.0).

### 3.6. Machine Learning Modeling

The gradient boosting (GB) model, a tree-based ensemble machine learning algorithm, was developed using Scikit-learn (v0.24.2) in Python (v3.8.12) to classify different types of Tieguanyin oolong tea. The hyperparameters were optimized through GridSearchCV. The 133 light-scented tea samples were analyzed using all 181 identified volatile compounds as features. The samples were stratified and randomly split, with 80% (106 samples, including 58 spring and 48 autumn) for training and 20% (27 samples, including 15 spring and 12 autumn) for external validation. The stratified sampling ensured the proportional representation of both seasons in the validation set. Model performance was assessed using five-fold cross-validation on the training set. Multiple evaluation metrics were employed, including accuracy, precision, recall, receiver operating characteristic (ROC) curves, and area under the curve (AUC). Additionally, SHapley Additive exPlanations (SHAP) analysis was conducted to quantify the relative contributions of individual metabolites to the classification model.

## Data Availability

Data are contained within the article.

## Supplementary Materials

The following supporting information can be downloaded at: https://www.mdpi.com/article/10.3390/plants14152378/s1. Figure S1: PCA plot for Tieguanyin oolong tea made from leaves harvested in spring and autumn. A: light scented tea, B: strong scented tea; Figure S2: Premutation test of OPLSDA for light scented tea; Figure S3: A scheme of transformation between linalool related products; Table S1: Detailed information about collected samples; Table S2: List of seasonal differentiating metabolites for light-scented Tieguanyin tea made from spring and autumn harvested leaves; Table S3: List of sensory differentiating metabolites for light-scented Tieguanyin tea between high-quality (tope 30 in aroma score) and low quality (bottom 30 in aroma score).

## References

[1] Zhou P., Li Z., Ouyang L., Gong X., Meng P., Dai M., Wang Z., Wang Y. (2019). A multi-element stable isotope approach coupled with chemometrics for the determination of Tieguanyin tea geographical origin and harvest season. Anal. Methods.

[2] Zhou J., Gao S., Du Z., Jin S., Yang Z., Xu T., Zheng C., Liu Y. (2025). Seasonal variations and sensory profiles of oolong tea: Insights from metabolic analysis of Tieguanyin cultivar. Food Chem..

[3] Wang J., Li X., Wu Y., Qu F., Liu L., Wang B., Wang P., Zhang X. (2022). HS−SPME/GC−MS Reveals the Season Effects on Volatile Compounds of Green Tea in High−Latitude Region. Foods.

[4] Zhang C., Zhou C., Xu K., Tian C., Zhang M., Lu L., Zhu C., Lai Z., Guo Y. (2022). A Comprehensive Investigation of Macro-Composition and Volatile Compounds in Spring-Picked and Autumn-Picked White Tea. Foods.

[5] Yu P., Huang Y., Li Z., Zhao X., Huang H., Zhong N., Zheng H., Chen Q. (2023). Difference in Aroma Components of Black Teas Processed on Different Dates in the Spring Season. Foods.

[6] Gianturco M.A., Biggers R.E., Ridling B.H. (1974). Seasonal variations in the composition of the volatile constituents of black tea. A numerical approach to the correlation between composition and quality of tea aroma. J. Agric. Food Chem..

[7] Zeng L., Fu Y., Huang J., Wang J., Jin S., Yin J., Xu Y. (2022). Comparative analysis of volatile vompounds in Tieguanyin with different types based on HS-SPME-GC-MS. Foods.

[8] Zeng L., Fu Y.Q., Liu Y.Y., Huang J.S., Chen J.X., Yin J.F., Jin S., Sun W.J., Xu Y.Q. (2023). Comparative analysis of different grades of Tieguanyin oolong tea based on metabolomics and sensory evaluation. LWT-Food Sci. Technol..

[9] Zhou Y., He W., He Y., Chen Q., Gao Y., Geng J., Zhu Z.-R. (2023). Formation of 8-hydroxylinalool in tea plant *Camellia sinensis* var. Assamica ‘Hainan dayezhong’. Food Chem. Mol. Sci..

[10] Gui J., Fu X., Zhou Y., Katsuno T., Mei X., Deng R., Xu X., Zhang L., Dong F., Watanabe N. (2015). Does Enzymatic Hydrolysis of Glycosidically Bound Volatile Compounds Really Contribute to the Formation of Volatile Compounds During the Oolong Tea Manufacturing Process?. J. Agric. Food Chem..

[11] Scott E.R., Li X., Wei J.-P., Kfoury N., Morimoto J., Guo M., Agyei A., Robbat A., Ahmed S., Cash S.B. (2020). Changes in Tea Plant Secondary Metabolite Profiles as a Function of Leafhopper Density and Damage. Front. Plant Sci..

[12] Zeng L., Watanabe N., Yang Z. (2019). Understanding the biosyntheses and stress response mechanisms of aroma compounds in tea (*Camellia sinensis*) to safely and effectively improve tea aroma. Crit. Rev. Food Sci. Nutr..

[13] Wang M., Li J., Liu X., Liu C., Qian J., Yang J., Zhou X., Jia Y., Tang J., Zeng L. (2022). Characterization of Key Odorants in Lingtou Dancong Oolong Tea and Their Differences Induced by Environmental Conditions from Different Altitudes. Metabolites.

[14] Chen P., Cai J., Zheng P., Yuan Y., Tsewang W., Chen Y., Xiao X., Liao J., Sun B., Liu S. (2022). Quantitatively Unravelling the Impact of High Altitude on Oolong Tea Flavor from *Camellia sinensis* Grown on the Plateaus of Tibet. Horticulturae.

[15] Gouinguené S.P., Turlings T.C.J. (2002). The Effects of Abiotic Factors on Induced Volatile Emissions in Corn Plants. Plant Physiol..

[16] Rawat R., Gulati A. (2007). Seasonal and clonal variations in some major glycosidic bound volatiles in Kangra tea (*Camellia sinensis* (L.) O. Kuntze). Eur. Food Res. Technol..

[17] Ge X., Sun J., Lu B., Chen Q., Xun W., Jin Y. (2019). Classification of oolong tea varieties based on hyperspectral imaging technology and BOSS-LightGBM model. J. Food Process Eng..

[18] Li Y., Sun J., Wu X., Lu B., Wu M., Dai C. (2019). Grade Identification of Tieguanyin Tea Using Fluorescence Hyperspectra and Different Statistical Algorithms. J. Food Sci..

[19] Liu P., Wen Y., Huang J., Xiong A., Wen J., Li H., Huang Y., Zhu X., Ai S., Wu R. (2019). A novel strategy of near-infrared spectroscopy dimensionality reduction for discrimination of grades, varieties and origins of green tea. Vib. Spectrosc..

[20] Jiang J., Wang R., Wang M., Gao K., Duc Duy N., Wei G.-W. (2020). Boosting Tree-Assisted Multitask Deep Learning for Small Scientific Datasets. J. Chem. Inf. Model..

[21] Peng Y., Zheng C., Guo S., Gao F., Wang X., Du Z., Gao F., Su F., Zhang W., Yu X. (2023). Metabolomics integrated with machine learning to discriminate the geographic origin of Rougui Wuyi rock tea. NPJ Sci. Food.

[22] Jin J., Zhao M., Jing T., Zhang M., Lu M., Yu G., Wang J., Guo D., Pan Y., Hoffmann T.D. (2023). Volatile compound-mediated plant–plant interactions under stress with the tea plant as a model. Hortic. Res..

[23] Liu H., Xu Y., Wu J., Wen J., Yu Y., An K., Zou B. (2021). GC-IMS and olfactometry analysis on the tea aroma of Yingde black teas harvested in different seasons. Food Res. Int..

[24] Zhu J., Chen F., Wang L., Niu Y., Xiao Z. (2017). Evaluation of the synergism among volatile compounds in Oolong tea infusion by odour threshold with sensory analysis and E-nose. Food Chem..

[25] Ito Y., Kubota K. (2005). Sensory evaluation of the synergism among odorants present in concentrations below their odor threshold in a Chinese jasmine green tea infusion. Mol. Nutr. Food Res..

[26] He C., Zhou J., Li Y., Zhang D., Ntezimana B., Zhu J., Wang X., Xu W., Wen X., Chen Y. (2023). The aroma characteristics of oolong tea are jointly determined by processing mode and tea cultivars. Food Chem..

[27] He C., Li Y., Zhou J., Yu X., Zhang D., Chen Y., Ni D., Yu Z. (2022). Study on the Suitability of Tea Cultivars for Processing Oolong Tea from the Perspective of Aroma Based on Olfactory Sensory, Electronic Nose, and GC-MS Data Correlation Analysis. Foods.

[28] Baldermann S., Yang Z., Katsuno T., Tu V.A., Mase N., Nakamura Y., Watanabe N. (2014). Discrimination of Green, Oolong, and Black Teas by GC-MS Analysis of Characteristic Volatile Flavor Compounds. Am. J. Anal. Chem..

[29] Lin J., Dai Y., Guo Y.n., Xu H., Wang X. (2012). Volatile profile analysis and quality prediction of Longjing tea (*Camellia sinensis*) by HS-SPME/GC-MS. J. Zhejiang Univ. Sci. B.

[30] Chen S., Xie P., Li Y., Wang X., Liu H., Wang S., Han W., Wu R., Li X., Guan Y. (2021). New Insights into Stress-Induced β-Ocimene Biosynthesis in Tea (*Camellia sinensis*) Leaves during Oolong Tea Processing. J. Agric. Food Chem..

[31] Peng Y., Du Z., Wang X., Wu R., Zheng C., Han W., Liu L., Gao F., Liu G., Liu B. (2024). From heat to flavor: Unlocking new chemical signatures to discriminate Wuyi rock tea under light and moderate roasting. Food Chem..

[32] (2013). Oolong Tea—Part 2: Tieguanyin.

[33] Worley B., Powers R. (2013). Multivariate Analysis in Metabolomics. Curr. Metabolomics.

