# Supplementary material for: The Impact of Harvest Season on Oolong Tea Aroma Profile and Quality
Source: Plants (Basel). 2025 Aug 1;14(15):2378. doi: 10.3390/plants14152378 (PMC12349564; doi:10.3390/plants14152378)
Supplement: Supplementary file 1 [file plants-14-02378-s001.zip › plants-3766391-supplementary.pdf]

The Impact of Harvest Season on Oolong Tea Aroma Profile and Quality

Chao Zheng[1], Shuilian Gao[2], Xiaxia Wang[1], Zhenbiao Yang[1], Junling Zhou[1,*], Ying Liu[1,*]

[1], Haixia Institute of Science and Technology, Fujian Agriculture and Forestry University, Fuzhou, 350007, China.

[2], Anxi College of Tea Science, Fujian Agriculture and Forestry University, Fuzhou, 350007, China.

*Corresponding author

Junling Zhou: junling-zhou@fafu.edu.cn
Ying Liu: ying.liu@fafu.edu.cn

Supplementary Table S1. Detailed information about collected samples.

ID	Type	Tea Companies	Price Ranges	Production Time	Aroma score
21Q01	Light-scented	Anxi Tieguanyin Co., Ltd	200RMB<B≦500RMB	Spring, 2021	86
21Q02	Light-scented	Anxi Tieguanyin Co., Ltd	1000RMB<D≦3000RMB	Spring, 2021	98
21Q03	Light-scented	Anxi Tieguanyin Co., Ltd	1000RMB<D≦3000RMB	Spring, 2021	105
21Q04	Light-scented	Anxi Tieguanyin Co., Ltd	E>3000RMB	Spring, 2021	105
21Q05	Light-scented	Xin Bai He Tea Co., Ltd	A≦200RMB	Spring, 2021	76
21Q06	Light-scented	Xin Bai He Tea Co., Ltd	200RMB<B≦500RMB	Spring, 2021	88
21Q07	Light-scented	Xin Bai He Tea Co., Ltd	500RMB<C≦1000RMB	Spring, 2021	99
21Q08	Light-scented	Mu Yun Shan Tea Co., Ltd	A≦200RMB	Spring, 2021	76
21Q09	Light-scented	Mu Yun Shan Tea Co., Ltd	200RMB<B≦500RMB	Spring, 2021	83
21Q10	Light-scented	Mu Yun Shan Tea Co., Ltd	1000RMB<D≦3000RMB	Spring, 2021	105
21Q11	Light-scented	Xiang Min Tea Co., Ltd	200RMB<B≦500RMB	Spring, 2021	85
21Q12	Light-scented	Xiang Min Tea Co., Ltd	E>3000RMB	Spring, 2021	102
21Q13	Light-scented	Da Zi Ran Tea Co., Ltd	200RMB<B≦500RMB	Spring, 2021	88
21Q14	Light-scented	Da Zi Ran Tea Co., Ltd	500RMB<C≦1000RMB	Spring, 2021	88
21Q15	Light-scented	Da Zi Ran Tea Co., Ltd	500RMB<C≦1000RMB	Spring, 2021	94
21Q16	Light-scented	Anxi Tea Market	A≦200RMB	Spring, 2021	76
21Q17	Light-scented	Anxi Tea Market	A≦200RMB	Spring, 2021	72
21Q18	Light-scented	Anxi Tea Market	A≦200RMB	Spring, 2021	70
21Q19	Light-scented	Anxi Tea Market	A≦200RMB	Spring, 2021	74
21Q20	Light-scented	Anxi Tea Market	A≦200RMB	Spring, 2021	74

21Q21	Light-scented	Anxi Tea Market	A≦200RMB	Spring, 2021	75
21Q22	Light-scented	Anxi Tea Market	A≦200RMB	Spring, 2021	73
21Q23	Light-scented	Chen Su Quan Tea Co., Ltd	200RMB<B≦500RMB	Spring, 2021	84
21Q24	Light-scented	Chen Su Quan Tea Co., Ltd	200RMB<B≦500RMB	Spring, 2021	89
21Q25	Light-scented	Chen Su Quan Tea Co., Ltd	500RMB<C≦1000RMB	Spring, 2021	98
21Q26	Light-scented	Chen Su Quan Tea Co., Ltd	1000RMB<D≦3000RMB	Spring, 2021	104
21Q27	Light-scented	Chen Su Quan Tea Co., Ltd	1000RMB<D≦3000RMB	Spring, 2021	103
21Q29	Light-scented	Chen Su Quan Tea Co., Ltd	E>3000RMB	Spring, 2021	101
21Q30	Light-scented	Chen Su Quan Tea Co., Ltd	E>3000RMB	Spring, 2021	106
21Q31	Light-scented	Guan He Tea Co., Ltd	A≦200RMB	Spring, 2021	81
21Q32	Light-scented	Guan He Tea Co., Ltd	200RMB<B≦500RMB	Spring, 2021	90
21Q33	Light-scented	Guan He Tea Co., Ltd	500RMB<C≦1000RMB	Spring, 2021	98
21Q34	Light-scented	Guan He Tea Co., Ltd	1000RMB<D≦3000RMB	Spring, 2021	103
21Q35	Light-scented	Guan He Tea Co., Ltd	E>3000RMB	Spring, 2021	104
21Q36	Light-scented	Guan He Tea Co., Ltd	1000RMB<D≦3000RMB	Spring, 2021	108
21Q37	Light-scented	Hua Yuan Tea Co., Ltd	500RMB<C≦1000RMB	Spring, 2021	91
21Q38	Light-scented	Hua Yuan Tea Co., Ltd	500RMB<C≦1000RMB	Spring, 2021	100
21Q39	Light-scented	Hua Yuan Tea Co., Ltd	1000RMB<D≦3000RMB	Spring, 2021	100
21Q40	Light-scented	Hua Yuan Tea Co., Ltd	E>3000RMB	Spring, 2021	103
21Q41	Light-scented	Hua Yuan Tea Co., Ltd	200RMB<B≦500RMB	Spring, 2021	87
21Q42	Light-scented	Shuang Suan Tea Co., Ltd	200RMB<B≦500RMB	Spring, 2021	90
21Q43	Light-scented	Shuang Suan Tea Co., Ltd	500RMB<C≦1000RMB	Spring, 2021	101

21Q44	Light-scented	Shuang Suan Tea Co., Ltd	200RMB<B≦500RMB	Spring, 2021	87
21Q45	Light-scented	Xin Yu Tea Co., Ltd	200RMB<B≦500RMB	Spring, 2021	85
21Q46	Light-scented	Xin Yu Tea Co., Ltd	200RMB<B≦500RMB	Spring, 2021	88
21Q47	Light-scented	Xin Yu Tea Co., Ltd	1000RMB<D≦3000RMB	Spring, 2021	100
21Q48	Light-scented	Cha Si Zhang Tea Co., Ltd	A≦200RMB	Spring, 2021	78
21Q49	Light-scented	Cha Si Zhang Tea Co., Ltd	200RMB<B≦500RMB	Spring, 2021	86
21Q50	Light-scented	Cha Si Zhang Tea Co., Ltd	1000RMB<D≦3000RMB	Spring, 2021	100
21Q51	Light-scented	Yu Cha Gong Wu Tea Co., Ltd	A≦200RMB	Spring, 2021	82
21Q52	Light-scented	Yu Cha Gong Wu Tea Co., Ltd	200RMB<B≦500RMB	Spring, 2021	81
21Q53	Light-scented	Yu Cha Gong Wu Tea Co., Ltd	500RMB<C≦1000RMB	Spring, 2021	91
21Q55	Light-scented	Zheng Shan Fu Tea Co., Ltd	200RMB<B≦500RMB	Spring, 2021	82
21Q56	Light-scented	Zheng Shan Fu Tea Co., Ltd	200RMB<B≦500RMB	Spring, 2021	93
21Q57	Light-scented	Wan Nian Fang Tea Co., Ltd	A≦200RMB	Spring, 2021	79
21Q58	Light-scented	Wan Nian Fang Tea Co., Ltd	200RMB<B≦500RMB	Spring, 2021	82
21Q59	Light-scented	Wan Nian Fang Tea Co., Ltd	500RMB<C≦1000RMB	Spring, 2021	90
21Q60	Light-scented	Nian Nian Xiang Tea Co., Ltd	A≦200RMB	Spring, 2021	72
21Q61	Light-scented	Nian Nian Xiang Tea Co., Ltd	A≦200RMB	Spring, 2021	81
21Q62	Light-scented	Nian Nian Xiang Tea Co., Ltd	200RMB<B≦500RMB	Spring, 2021	82
21Q63	Light-scented	Nian Nian Xiang Tea Co., Ltd	200RMB<B≦500RMB	Spring, 2021	88
21Q64	Light-scented	Nian Nian Xiang Tea Co., Ltd	500RMB<C≦1000RMB	Spring, 2021	86
21Q65	Light-scented	Nian Nian Xiang Tea Co., Ltd	500RMB<C≦1000RMB	Spring, 2021	94
21Q66	Light-scented	Nian Nian Xiang Tea Co., Ltd	1000RMB<D≦3000RMB	Spring, 2021	99

21Q67	Light-scented	Anxi Sanhe Tea Co., Ltd	200RMB<B≦500RMB	Spring, 2021	87
21Q68	Light-scented	Anxi Sanhe Tea Co., Ltd	500RMB<C≦1000RMB	Spring, 2021	94
21Q69	Light-scented	Anxi Sanhe Tea Co., Ltd	1000RMB<D≦3000RMB	Spring, 2021	102
21Q73	Light-scented	Anxi Fanwu Tea Co., Ltd	1000RMB<D≦3000RMB	Spring, 2021	80
21Q74	Light-scented	Anxi Fanwu Tea Co., Ltd	500RMB<C≦1000RMB	Spring, 2021	76
21Q75	Light-scented	Anxi Fanwu Tea Co., Ltd	500RMB<C≦1000RMB	Spring, 2021	96
21Q77	Light-scented	Fujian Bama Tea Co., Ltd	500RMB<C≦1000RMB	Spring, 2021	94
21Q83	Light-scented	Anxi Tieguanyin Co., Ltd	A≦200RMB	Spring, 2021	73
21Q84	Light-scented	Anxi Tieguanyin Co., Ltd	A≦200RMB	Spring, 2021	83
20Q01	Light-scented	Anxi Aimin Tea Co., Ltd	A≦200RMB	Autumn, 2020	93
20Q04	Light-scented	Fujian Bama Tea Co., Ltd	200RMB<B≦500RMB	Autumn, 2020	81
20Q05	Light-scented	Fujian Bama Tea Co., Ltd	500RMB<C≦1000RMB	Autumn, 2020	83
20Q06	Light-scented	Fujian Bama Tea Co., Ltd	1000RMB<D≦3000RMB	Autumn, 2020	102
20Q07	Light-scented	Dong Keng Gong Ying Tea Specialized Cooperative	A≦200RMB	Autumn, 2020	91
20Q08	Light-scented	Dong Keng Gong Ying Tea Specialized Cooperative	200RMB<B≦500RMB	Autumn, 2020	93
20Q09	Light-scented	Dong Keng Gong Ying Tea Specialized Cooperative	1000RMB<D≦3000RMB	Autumn, 2020	101
20Q10	Light-scented	Dong Keng Gong Ying Tea Specialized Cooperative	1000RMB<D≦3000RMB	Autumn, 2020	103
20Q11	Light-scented	Hua Yuan Tea Co., Ltd	A≦200RMB	Autumn, 2020	92

20Q12	Light-scented	Hua Yuan Tea Co., Ltd	200RMB<B≦500RMB	Autumn, 2020	91
20Q13	Light-scented	Hua Yuan Tea Co., Ltd	200RMB<B≦500RMB	Autumn, 2020	96
20Q14	Light-scented	Hua Yuan Tea Co., Ltd	500RMB<C≦1000RMB	Autumn, 2020	93
20Q15	Light-scented	Hua Yuan Tea Co., Ltd	1000RMB<D≦3000RMB	Autumn, 2020	94
20Q16	Light-scented	Quanzhou Ji Yan Tea Co., Ltd	A≦200RMB	Autumn, 2020	83
20Q17	Light-scented	Quanzhou Ji Yan Tea Co., Ltd	200RMB<B≦500RMB	Autumn, 2020	91
20Q18	Light-scented	Quanzhou Ji Yan Tea Co., Ltd	200RMB<B≦500RMB	Autumn, 2020	87
20Q19	Light-scented	Quanzhou Ji Yan Tea Co., Ltd	1000RMB<D≦3000RMB	Autumn, 2020	89
20Q20	Light-scented	Quanzhou Ji Yan Tea Co., Ltd	E>3000RMB	Autumn, 2020	85
20Q21	Light-scented	Shan Ren Tea Co., Ltd	A≦200RMB	Autumn, 2020	85
20Q22	Light-scented	Shan Ren Tea Co., Ltd	A≦200RMB	Autumn, 2020	89
20Q23	Light-scented	Shan Ren Tea Co., Ltd	200RMB<B≦500RMB	Autumn, 2020	89
20Q24	Light-scented	Shan Ren Tea Co., Ltd	500RMB<C≦1000RMB	Autumn, 2020	88
20Q25	Light-scented	Shan Ren Tea Co., Ltd	E>3000RMB	Autumn, 2020	87
20Q26	Light-scented	You Xiang Yuan Tea Co., Ltd	A≦200RMB	Autumn, 2020	90
20Q27	Light-scented	You Xiang Yuan Tea Co., Ltd	200RMB<B≦500RMB	Autumn, 2020	90
20Q28	Light-scented	You Xiang Yuan Tea Co., Ltd	500RMB<C≦1000RMB	Autumn, 2020	95
20Q29	Light-scented	You Xiang Yuan Tea Co., Ltd	1000RMB<D≦3000RMB	Autumn, 2020	91
20Q30	Light-scented	You Xiang Yuan Tea Co., Ltd	E>3000RMB	Autumn, 2020	99
20Q31	Light-scented	Anxi Xin Kang Tea Co., Ltd	200RMB<B≦500RMB	Autumn, 2020	88
20Q32	Light-scented	Anxi Xin Kang Tea Co., Ltd	500RMB<C≦1000RMB	Autumn, 2020	92
20Q33	Light-scented	Anxi Xin Kang Tea Co., Ltd	E>3000RMB	Autumn, 2020	97

20Q34	Light-scented	Da Zi Ran Tea Co., Ltd	A≦200RMB	Autumn, 2020	85
20Q35	Light-scented	Da Zi Ran Tea Co., Ltd	200RMB<B≦500RMB	Autumn, 2020	89
20Q36	Light-scented	Da Zi Ran Tea Co., Ltd	500RMB<C≦1000RMB	Autumn, 2020	93
20Q37	Light-scented	Da Zi Ran Tea Co., Ltd	1000RMB<D≦3000RMB	Autumn, 2020	91
20Q38	Light-scented	Anxi Tieguanyin Co., Ltd	A≦200RMB	Autumn, 2020	79
20Q39	Light-scented	Anxi Tieguanyin Co., Ltd	A≦200RMB	Autumn, 2020	79
20Q40	Light-scented	Anxi Tieguanyin Co., Ltd	1000RMB<D≦3000RMB	Autumn, 2020	91
20Q41	Light-scented	Anxi Tieguanyin Co., Ltd	200RMB<B≦500RMB	Autumn, 2020	91
20Q42	Light-scented	Anxi Tieguanyin Co., Ltd	1000RMB<D≦3000RMB	Autumn, 2020	94
20Q43	Light-scented	Anxi Tieguanyin Co., Ltd	1000RMB<D≦3000RMB	Autumn, 2020	103
20Q44	Light-scented	Anxi Tieguanyin Co., Ltd	E>3000RMB	Autumn, 2020	98
20Q52	Light-scented	Xin Qing Hong Tea Co., Ltd	A≦200RMB	Autumn, 2020	91
20Q53	Light-scented	Xin Qing Hong Tea Co., Ltd	200RMB<B≦500RMB	Autumn, 2020	95
20Q54	Light-scented	Xin Qing Hong Tea Co., Ltd	200RMB<B≦500RMB	Autumn, 2020	94
20Q55	Light-scented	Xin Qing Hong Tea Co., Ltd	500RMB<C≦1000RMB	Autumn, 2020	103
20Q56	Light-scented	Xin Qing Hong Tea Co., Ltd	1000RMB<D≦3000RMB	Autumn, 2020	103
20Q57	Light-scented	Xin Qing Hong Tea Co., Ltd	1000RMB<D≦3000RMB	Autumn, 2020	89
20Q58	Light-scented	Jin Heng Feng Tea Co., Ltd	A≦200RMB	Autumn, 2020	99
20Q59	Light-scented	Jin Heng Feng Tea Co., Ltd	200RMB<B≦500RMB	Autumn, 2020	100
20Q60	Light-scented	Jin Heng Feng Tea Co., Ltd	500RMB<C≦1000RMB	Autumn, 2020	103
20Q61	Light-scented	Ji Tie Tea Co., Ltd	A≦200RMB	Autumn, 2020	83
20Q62	Light-scented	Ji Tie Tea Co., Ltd	200RMB<B≦500RMB	Autumn, 2020	93

20Q66	Light-scented	Nian Nian Xiang Tea Co., Ltd	200RMB<B≦500RMB	Autumn, 2020	90
20Q68	Light-scented	Nian Nian Xiang Tea Co., Ltd	500RMB<C≦1000RMB	Autumn, 2020	94
20Q70	Light-scented	Nian Nian Xiang Tea Co., Ltd	1000RMB<D≦3000RMB	Autumn, 2020	92
21N01	Strong-scented	Anxi Tieguanyin Co., Ltd	500RMB<C≦1000RMB	Spring, 2021	86
21N02	Strong-scented	Anxi Tieguanyin Co., Ltd	1000RMB<D≦3000RMB	Spring, 2021	85
21N03	Strong-scented	Anxi Tieguanyin Co., Ltd	1000RMB<D≦3000RMB	Spring, 2021	105
21N04	Strong-scented	Anxi Tieguanyin Co., Ltd	E>3000RMB	Spring, 2021	103
21N05	Strong-scented	Liu Jin Long Co., Ltd	500RMB<C≦1000RMB	Spring, 2021	84
21N06	Strong-scented	Liu Jin Long Co., Ltd	1000RMB<D≦3000RMB	Spring, 2021	99
21N10	Strong-scented	Xiang Min Tea Co., Ltd	200RMB<B≦500RMB	Spring, 2021	83
21N11	Strong-scented	Xiang Min Tea Co., Ltd	200RMB<B≦500RMB	Spring, 2021	87
21N12	Strong-scented	Chen Su Quan Tea Co., Ltd	200RMB<B≦500RMB	Spring, 2021	87
21N13	Strong-scented	Chen Su Quan Tea Co., Ltd	200RMB<B≦500RMB	Spring, 2021	84
21N14	Strong-scented	Chen Su Quan Tea Co., Ltd	500RMB<C≦1000RMB	Spring, 2021	92
21N15	Strong-scented	Chen Su Quan Tea Co., Ltd	1000RMB<D≦3000RMB	Spring, 2021	93
21N16	Strong-scented	Chen Su Quan Tea Co., Ltd	1000RMB<D≦3000RMB	Spring, 2021	102
21N17	Strong-scented	Chen Su Quan Tea Co., Ltd	1000RMB<D≦3000RMB	Spring, 2021	84
21N18	Strong-scented	Chen Su Quan Tea Co., Ltd	E>3000RMB	Spring, 2021	103
21N19	Strong-scented	Chen Su Quan Tea Co., Ltd	E>3000RMB	Spring, 2021	106
21N20	Strong-scented	Hua Yuan Tea Co., Ltd	200RMB<B≦500RMB	Spring, 2021	88
21N21	Strong-scented	Hua Yuan Tea Co., Ltd	500RMB<C≦1000RMB	Spring, 2021	88
21N22	Strong-scented	Hua Yuan Tea Co., Ltd	500RMB<C≦1000RMB	Spring, 2021	98

21N23	Strong-scented	Hua Yuan Tea Co., Ltd	1000RMB<D≦3000RMB	Spring, 2021	101
21N24	Strong-scented	Hua Yuan Tea Co., Ltd	E>3000RMB	Spring, 2021	104
21N25	Strong-scented	Shuang Suan Tea Co., Ltd	200RMB<B≦500RMB	Spring, 2021	89
21N26	Strong-scented	Shuang Suan Tea Co., Ltd	200RMB<B≦500RMB	Spring, 2021	90
21N27	Strong-scented	Shuang Suan Tea Co., Ltd	1000RMB<D≦3000RMB	Spring, 2021	96
21N28	Strong-scented	Anxi Tea Market	A≦200RMB	Spring, 2021	73
21N29	Strong-scented	Anxi Tea Market	A≦200RMB	Spring, 2021	74
21N30	Strong-scented	Anxi Pin Xin Tea Co., Ltd	200RMB<B≦500RMB	Spring, 2021	81
21N31	Strong-scented	Yu Cha Gong Wu Tea Co., Ltd	200RMB<B≦500RMB	Spring, 2021	84
21N32	Strong-scented	Yu Cha Gong Wu Tea Co., Ltd	500RMB<C≦1000RMB	Spring, 2021	85
21N33	Strong-scented	Yun Ling Pu'er Tea Co., Ltd	200RMB<B≦500RMB	Spring, 2021	84
21N34	Strong-scented	Ji Tie Tea Co., Ltd	A≦200RMB	Spring, 2021	77
21N35	Strong-scented	Hua Cheng Tea Co., Ltd	A≦200RMB	Spring, 2021	75
21N36	Strong-scented	Hua Cheng Tea Co., Ltd	200RMB<B≦500RMB	Spring, 2021	81
21N37	Strong-scented	Hua Cheng Tea Co., Ltd	500RMB<C≦1000RMB	Spring, 2021	87
21N38	Strong-scented	Anxi Sanhe Tea Co., Ltd	A≦200RMB	Spring, 2021	79
21N39	Strong-scented	Anxi Sanhe Tea Co., Ltd	500RMB<C≦1000RMB	Spring, 2021	86
21N40	Strong-scented	Anxi Sanhe Tea Co., Ltd	500RMB<C≦1000RMB	Spring, 2021	91
21N44	Strong-scented	Lin Qing Lan Tea Co., Ltd	500RMB<C≦1000RMB	Spring, 2021	74
21N45	Strong-scented	Lin Qing Lan Tea Co., Ltd	1000RMB<D≦3000RMB	Spring, 2021	80
21N46	Strong-scented	Lin Qing Lan Tea Co., Ltd	500RMB<C≦1000RMB	Spring, 2021	88
21N47	Strong-scented	Liu Jin Long Co., Ltd	A≦200RMB	Spring, 2021	80

21N48	Strong-scented	Liu Jin Long Co., Ltd	200RMB<B≦500RMB	Spring, 2021	79
21N49	Strong-scented	Liu Jin Long Co., Ltd	200RMB<B≦500RMB	Spring, 2021	86
21N50	Strong-scented	Fujian Bama Tea Co., Ltd	A≦200RMB	Spring, 2021	72
21N51	Strong-scented	Fujian Bama Tea Co., Ltd	A≦200RMB	Spring, 2021	79
21N52	Strong-scented	Fujian Bama Tea Co., Ltd	1000RMB<D≦3000RMB	Spring, 2021	91
21N53	Strong-scented	Fujian Bama Tea Co., Ltd	E>3000RMB	Spring, 2021	104
21N54	Strong-scented	Fujian Bama Tea Co., Ltd	E>3000RMB	Spring, 2021	104
21N58	Strong-scented	Anxi Fanwu Tea Co., Ltd	A≦200RMB	Spring, 2021	82
21N59	Strong-scented	Tian Xiang Hun Co., Ltd	1000RMB<D≦3000RMB	Spring, 2021	100
21N60	Strong-scented	Tian Xiang Hun Co., Ltd	1000RMB<D≦3000RMB	Spring, 2021	104
21N61	Strong-scented	Tian Xiang Hun Co., Ltd	500RMB<C≦1000RMB	Spring, 2021	92
21N62	Strong-scented	Tian Xiang Hun Co., Ltd	500RMB<C≦1000RMB	Spring, 2021	83
21N63	Strong-scented	Tian Xiang Hun Co., Ltd	200RMB<B≦500RMB	Spring, 2021	81
21N64	Strong-scented	Anxi Tieguanyin Co., Ltd	A≦200RMB	Spring, 2021	74
21N65	Strong-scented	Li Shan Cha Xian Co., Ltd	A≦200RMB	Spring, 2021	77
21N66	Strong-scented	Li Shan Cha Xian Co., Ltd	A≦200RMB	Spring, 2021	77
21N67	Strong-scented	Li Shan Cha Xian Co., Ltd	200RMB<B≦500RMB	Spring, 2021	77
21N68	Strong-scented	Anxi Tea Market	A≦200RMB	Spring, 2021	82
21N69	Strong-scented	Anxi Tea Market	A≦200RMB	Spring, 2021	87
21N70	Strong-scented	Anxi Tea Market	A≦200RMB	Spring, 2021	83
21N71	Strong-scented	Anxi Tea Market	A≦200RMB	Spring, 2021	92
21N72	Strong-scented	Nian Nian Xiang Tea Co., Ltd	A≦200RMB	Spring, 2021	77

21N73	Strong-scented	Nian Nian Xiang Tea Co., Ltd	500RMB<C≦1000RMB	Spring, 2021	83
21N74	Strong-scented	Ju Yuan Tea Co., Ltd	200RMB<B≦500RMB	Spring, 2021	83
21N75	Strong-scented	Wei Yue De Tea Co., Ltd	A≦200RMB	Spring, 2021	75
20N01	Strong-scented	Anxi Tieguanyin Co., Ltd	A≦200RMB	Autumn, 2020	72
20N02	Strong-scented	Anxi Tieguanyin Co., Ltd	A≦200RMB	Autumn, 2020	77
20N03	Strong-scented	Anxi Tieguanyin Co., Ltd	200RMB<B≦500RMB	Autumn, 2020	94
20N04	Strong-scented	Anxi Tieguanyin Co., Ltd	1000RMB<D≦3000RMB	Autumn, 2020	106
20N05	Strong-scented	Anxi Tieguanyin Co., Ltd	1000RMB<D≦3000RMB	Autumn, 2020	105
20N06	Strong-scented	Anxi Tieguanyin Co., Ltd	E>3000RMB	Autumn, 2020	106
20N07	Strong-scented	Anxi Aimin Tea Co., Ltd	A≦200RMB	Autumn, 2020	80
20N08	Strong-scented	Anxi Xin Kang Tea Co., Ltd	A≦200RMB	Autumn, 2020	73
20N09	Strong-scented	Anxi Xin Kang Tea Co., Ltd	A≦200RMB	Autumn, 2020	80
20N10	Strong-scented	Anxi Xin Kang Tea Co., Ltd	500RMB<C≦1000RMB	Autumn, 2020	95
20N11	Strong-scented	Anxi Xin Kang Tea Co., Ltd	1000RMB<D≦3000RMB	Autumn, 2020	103
20N12	Strong-scented	Fujian Bama Tea Co., Ltd	A≦200RMB	Autumn, 2020	74
20N13	Strong-scented	Fujian Bama Tea Co., Ltd	200RMB<B≦500RMB	Autumn, 2020	89
20N14	Strong-scented	Fujian Bama Tea Co., Ltd	1000RMB<D≦3000RMB	Autumn, 2020	95
20N15	Strong-scented	Fujian Bama Tea Co., Ltd	1000RMB<D≦3000RMB	Autumn, 2020	104
20N16	Strong-scented	Fujian Bama Tea Co., Ltd	E>3000RMB	Autumn, 2020	103
20N17	Strong-scented	Fujian Bama Tea Co., Ltd	E>3000RMB	Autumn, 2020	104
20N18	Strong-scented	Fujian Bama Tea Co., Ltd	E>3000RMB	Autumn, 2020	104
20N19	Strong-scented	Hua Yuan Tea Co., Ltd	A≦200RMB	Autumn, 2020	86

20N20	Strong-scented	Hua Yuan Tea Co., Ltd	200RMB<B≦500RMB	Autumn, 2020	93
20N21	Strong-scented	Hua Yuan Tea Co., Ltd	200RMB<B≦500RMB	Autumn, 2020	97
20N22	Strong-scented	Hua Yuan Tea Co., Ltd	500RMB<C≦1000RMB	Autumn, 2020	101
20N23	Strong-scented	Quanzhou Ji Yan Tea Co., Ltd	A≦200RMB	Autumn, 2020	81
20N24	Strong-scented	Quanzhou Ji Yan Tea Co., Ltd	200RMB<B≦500RMB	Autumn, 2020	85
20N25	Strong-scented	Quanzhou Ji Yan Tea Co., Ltd	500RMB<C≦1000RMB	Autumn, 2020	94
20N26	Strong-scented	Quanzhou Ji Yan Tea Co., Ltd	1000RMB<D≦3000RMB	Autumn, 2020	97
20N27	Strong-scented	Dong Keng Gong Ying Tea Specialized Cooperative	1000RMB<D≦3000RMB	Autumn, 2020	102
20N28	Strong-scented	Dong Keng Gong Ying Tea Specialized Cooperative	A≦200RMB	Autumn, 2020	91
20N29	Strong-scented	Dong Keng Gong Ying Tea Specialized Cooperative	200RMB<B≦500RMB	Autumn, 2020	92
20N30	Strong-scented	Xin Bai He Tea Co., Ltd	A≦200RMB	Autumn, 2020	81
20N31	Strong-scented	Xin Bai He Tea Co., Ltd	200RMB<B≦500RMB	Autumn, 2020	90
20N32	Strong-scented	Xin Bai He Tea Co., Ltd	500RMB<C≦1000RMB	Autumn, 2020	100
20N33	Strong-scented	Mu Yun Shan Tea Co., Ltd	200RMB<B≦500RMB	Autumn, 2020	91
20N34	Strong-scented	Mu Yun Shan Tea Co., Ltd	E>3000RMB	Autumn, 2020	106
20N35	Strong-scented	Da Zi Ran Tea Co., Ltd	500RMB<C≦1000RMB	Autumn, 2020	100
20N36	Strong-scented	Da Zi Ran Tea Co., Ltd	500RMB<C≦1000RMB	Autumn, 2020	94
20N37	Strong-scented	Da Zi Ran Tea Co., Ltd	500RMB<C≦1000RMB	Autumn, 2020	87
20N38	Strong-scented	Da Zi Ran Tea Co., Ltd	E>3000RMB	Autumn, 2020	100

20N39	Strong-scented	Da Zi Ran Tea Co., Ltd	1000RMB<D≦3000RMB	Autumn, 2020	99
20N40	Strong-scented	Anxi Tea Market	A≦200RMB	Autumn, 2020	77
20N41	Strong-scented	Anxi Tea Market	A≦200RMB	Autumn, 2020	73
20N42	Strong-scented	Anxi Pin Xin Tea Co., Ltd	200RMB<B≦500RMB	Autumn, 2020	81
20N43	Strong-scented	Ji Tie Tea Co., Ltd	500RMB<C≦1000RMB	Autumn, 2020	99
20N44	Strong-scented	Ji Tie Tea Co., Ltd	500RMB<C≦1000RMB	Autumn, 2020	88
20N45	Strong-scented	Xin Yu Tea Co., Ltd	A≦200RMB	Autumn, 2020	86
20N46	Strong-scented	Xin Yu Tea Co., Ltd	200RMB<B≦500RMB	Autumn, 2020	93
20N47	Strong-scented	Xin Yu Tea Co., Ltd	500RMB<C≦1000RMB	Autumn, 2020	99
20N48	Strong-scented	Wan Nian Fang Tea Co., Ltd	A≦200RMB	Autumn, 2020	76
20N49	Strong-scented	Wan Nian Fang Tea Co., Ltd	200RMB<B≦500RMB	Autumn, 2020	82
20N50	Strong-scented	Wan Nian Fang Tea Co., Ltd	200RMB<B≦500RMB	Autumn, 2020	89
20N51	Strong-scented	Anxi Fanwu Tea Co., Ltd	200RMB<B≦500RMB	Autumn, 2020	97
20N52	Strong-scented	Anxi Fanwu Tea Co., Ltd	200RMB<B≦500RMB	Autumn, 2020	94
20N53	Strong-scented	Anxi Fanwu Tea Co., Ltd	200RMB<B≦500RMB	Autumn, 2020	96
20N54	Strong-scented	Anxi Fanwu Tea Co., Ltd	200RMB<B≦500RMB	Autumn, 2020	92
20N55	Strong-scented	Anxi Fanwu Tea Co., Ltd	A≦200RMB	Autumn, 2020	98
20N56	Strong-scented	Anxi Tea Market	200RMB<B≦500RMB	Autumn, 2020	91
20N57	Strong-scented	Anxi Tea Market	A≦200RMB	Autumn, 2020	86
20N58	Strong-scented	Anxi Tea Market	200RMB<B≦500RMB	Autumn, 2020	94
20N59	Strong-scented	Anxi Tea Market	200RMB<B≦500RMB	Autumn, 2020	85
20N60	Strong-scented	Anxi Tea Market	200RMB<B≦500RMB	Autumn, 2020	89

20N61	Strong-scented	Anxi Tea Market	A≦200RMB	Autumn, 2020	95
20N62	Strong-scented	Nian Nian Xiang Tea Co., Ltd	1000RMB<D≦3000RMB	Autumn, 2020	85
20N63	Strong-scented	Nian Nian Xiang Tea Co., Ltd	E>3000RMB	Autumn, 2020	84
20N64	Strong-scented	Wei Yue De Tea Co., Ltd	200RMB<B≦500RMB	Autumn, 2020	75
20N65	Strong-scented	Wei Yue De Tea Co., Ltd	500RMB<C≦1000RMB	Autumn, 2020	78
20N66	Strong-scented	Wei Yue De Tea Co., Ltd	500RMB<C≦1000RMB	Autumn, 2020	76
20N67	Strong-scented	Wei Yue De Tea Co., Ltd	1000RMB<D≦3000RMB	Autumn, 2020	88

Supplementary Table S2. List of seasonal differentiating metabolites for light-scented Tieguanyin tea made from spring and autumn harvested leaves.

Compounds	CAS No.	Formula	Metabolite Class	RT	Odor description	Category	RIexp	RIlit	Vip	p.value	FDR	Identified Method
trans-Linalool oxide (furanoid)	34995-77-2	C10H18O2	Heterocyclic compounds	1006.02	Floral	Floral	1078	1065-1098	2.83	6.79 E-21	2.10 E-18	MS, RI
Methyl salicylate	119-36-8	C8H8O3	Esters	1313.09	Seeet, spicy, minty	Sweet	1182	1188-1234	2.66	3.44 E-20	5.31 E-18	MS, RI, Std
trans-linalool oxide (pyranoid)	39028-58-5	C10H18O2	Heterocyclic compounds	1252.88	Woody, tea-like	Woody	1162	1162-1173	2.55	1.44 E-19	1.49 E-17	MS, RI
Benzoic acid, 2-methoxy-, methyl ester	606-45-1	C9H10O3	Esters	1716.43	Sweet, floral, and fruity	Sweet, Floral, Fruity	1328	1335-1336	2.51	2.23 E-19	1.72 E-17	MS, RI
Diendiol I	13741-21-4	C10H18O2	Alcohols	1327.57	Unknown	unknown	1187	1176-1186	2.33	2.34 E-16	1.20 E-14	MS, RI
Hotrienol	29957-43-5	C8H12O	Terpenoids	1060.61	Floral, green, woody	Floral, Green, Woody	1096	1107	2.23	2.32 E-15	8.95 E-14	MS, RI
Ethanol, 2-(pentyloxy)-, acetate	5312-09-4	C9H18O3	Esters	1365.75	Fruity and sweet	Fruity, Sweet	1200	1216	2.23	1.18 E-12	3.04 E-11	MS, RI
Linalool	78-70-6	C10H18O	Alcohols	1050.29	Floral odor	Floral	1093	1080-1110	2.16	5.49 E-15	1.88 E-13	MS, RI, Std
Isophytol	505-32-8	C20H40O	Terpenoids	3090.54	Mild, waxy, odorless	Little Odor	1934	1938-1950	2.08	1.55 E-10	2.67 E-09	MS, RI
2-Phenylethyl hexanoate	6290-37-5	C14H20O2	Esters	2452.67	Sweet, honey, floral	Sweet, floral	1629	1643-1649	2.07	1.01 E-10	1.84 E-09	MS, RI, Std

Neophytadiene	504-96-1	$C_{20}H_{38}$	Terpenoids	2873.65	Mild, woody, slightly citrusy	Woody, Fruity	1825	1836-1838	1.93	2.04 E-09	2.52 E-08	MS, RI
Benzyl nitrile	140-29-4	C_8H_7N	Aromatic compounds	1156.76	Mild, sweet, and almond-like	Sweet, Nutty	1129	1135-1160	1.85	1.56 E-09	2.01 E-08	MS, RI, Std
Phenethyl butyrate	103-52-6	$C_{12}H_{16}O_2$	Esters	1977.03	Sweet floral	Sweet, Floral	1429	1439-1447	1.85	3.74 E-09	4.13 E-08	MS, RI, Std
1-Octen-3-one	4312-99-6	$C_8H_{14}O$	Ketones	681.519	Mushroom-like	Pungent	969	962-982/976	1.83	4.19 E-12	9.24 E-11	MS, RI
(E)-4,8-Dimethylnona-1,3,7-triene	19945-61-0	$C_{11}H_{18}$	Terpenoids	1088.84	Spicy, woody, and slightly herbal	Woody	1106	1113	1.83	9.02 E-16	3.98 E-14	MS, RI
n-Caproic acid vinyl ester	3050-69-9	$C_8H_{14}O_2$	Esters	1504.92	Fruity, pineapple-like, slightly pungent	Fruity	1250	1244	1.81	2.46 E-08	2.38 E-07	MS, RI
1-Dodecyne	765-03-7	$C_{12}H_{22}$	others	2923.02	Mild, sweet	Sweet	1849	1836	1.81	2.33 E-09	2.77 E-08	MS, RI
2-Cyclopenten-1-one, 3-methyl-2-(2-pentenyl)-, (Z)-	488-10-8	$C_{11}H_{16}O$	Ketones	1862.67	Floral, jasmine, woody, herbal	Floral, Woody	1384	1392-1404	1.80	7.97 E-10	1.12 E-08	MS, RI
Phenylethyl butyrate	103-52-6	$C_{12}H_{16}O_2$	Esters	1859.56	fruit, pleasant	Fruity	1382	1396	1.76	6.69 E-08	6.08 E-07	MS, RI
Benzeneacetaldehyde	122-78-1	C_8H_8O	Aldehydes	874.268	Green floral and sweet	Green, Sweet	1035	1041/1049-1057	1.72	1.38 E-08	1.42 E-07	MS, RI, Std
1,7-Octadiene-3,6-diol, 2,6-dimethyl-	51276-33-6	$C_{10}H_{18}O_2$	Alcohols	1566.07	Floral, green, slightly fruity	Floral, Green	1272	1270-1277	1.72	7.45 E-12	1.53 E-10	MS, RI

Oxalic acid, isobutyl octyl ester	5779-95-3	C9H10O	Esters	1269.97	Unknown	unknown	1168	1169	1.68	3.45 E-17	2.13 E-15	MS, RI
Undecane, 2-methyl-	7045-71-8	C12H26	Alkanes	1240.53	Little to no odor	Little odor	1158	1163-1165	1.63	7.02 E-08	6.20 E-07	MS, RI
Benzene, (2-nitroethyl)-	6125-24-2	C8H9NO2	Aromatic compounds	1614.55	Floral, spicy	Floral, Spicy	1290	1300-1304	1.58	3.69 E-07	2.72 E-06	MS, RI
5-Hexen-2-one, 5-methyl-3-methylene-	51756-18-4	C7H12	Ketones	427.425	Sharp, sweet, fruity, green	Sweet, Fruity, Green	874	874-886	1.58	1.01 E-06	6.35 E-06	MS, RI
Benzaldehyde, 4-methyl-	104-87-0	C8H8O	Aldehydes	993.177	Strong, almond-like	Nutty	1074	1080	1.56	2.44 E-07	1.89 E-06	MS, RI
2-Furanmethanol, 5-ethenyltetrahydro-??5-trimethyl-, cis-	5989-33-3	C8H12O	Heterocyclic compounds	957.846	Woody, flowery	Woody, Floral	1062	1070-1087	1.52	3.63 E-14	1.12 E-12	MS, RI
1-Octanol	111-87-5	C21H21ClN2O8	Alcohols	971.582	Penetrating Aromatic	Little odor	1067	1063-1079	1.52	8.41 E-10	1.13 E-08	MS, RI, Std
Butanoic acid, 2-methyl-, 2-phenylethyl ester	24817-51-4	C13H18O2	Esters	2089.86	Fruity, sweet, slightly floral	Fruity, Sweet	1474	1484-1491	1.51	9.49 E-06	4.38 E-05	MS, RI
2(4H)-Benzofuranone, 5,6,7,7a-tetrahydro-4,4,7a-trimethyl-, (R)-	17092-92-1	C11H16O2	Heterocyclic compounds	2192.3	Musty, pungent	Pungent	1516	1518	1.49	1.97 E-06	1.15 E-05	MS, RI
1-Undecene	821-95-4	C11H22	Alkanes	976.738	Little to no odor	Little odor	1069	1088-1091	1.44	9.88 E-06	4.49 E-05	MS, RI

Cyclohexene, 4-[(1E)-1,5-dimethyl-1,4-hexadien-1-yl]-1-methyl-	25532-79-0	C15H24	Terpenoids	2223.41	sweet, floral, fruit	Sweet, Floral, Fruity	1530	1512	1.43	9.49 E-06	4.38 E-05	MS, RI
2-Heptenal, (Z)-	57266-86-1	C7H12O	Aldehydes	623.962	Green, fruity, grassy, cucumber-like	Green, Fruity	949	952-963/963	1.38	2.34 E-06	1.31 E-05	MS, RI
(E)-2-Octenal	2548-87-0	C8H14O	Aldehydes	923.008	Fatty, green aroma	Green	1051	1058	1.38	1.18 E-05	5.21 E-05	MS, RI, Std
3-Buten-2-one, 4-(2,2,6-trimethyl-7-oxabicyclo[4.1.0]hept-1-yl)-	23267-57-4	C13H20O2	Heterocyclic compounds	2081.8	woody, earthy,	Woody	1471	1455	1.35	7.55 E-05	2.95 E-04	MS, RI
Isopentyl hexanoate	2198-61-0	C11H22O2	Esters	1483.11	fruity, banana-like	Fruity, Sweet	1243	1244-1254	1.33	5.85 E-05	2.32 E-04	MS, RI
Benzaldehyde	100-52-7	C6H6O2	Aldehydes	634.016	Bitter almond, cherry	Nutty	952	955-962	1.32	4.38 E-06	2.18 E-05	MS, RI
Benzoic acid, 2-phenylethyl ester	94-47-3	C15H14O2	Esters	2909.87	Sweet, floral, slightly balsamic	Sweet, Floral	1843	1844-1858/1844	1.31	2.89 E-06	1.56 E-05	MS, RI
Octanal	124-13-0	C8H16O	Aldehydes	757.914	Strong, fruity	Fruity	996	1001/1004	1.30	3.35 E-05	1.36 E-04	MS, RI, Std
6-Methyl-6-(5-methylfuran-2-yl)heptan-2-one	50464-95-4	C13H20O2	Aromatic compounds	1928.35	Unknown	unknown	1409	1426-1439	1.30	1.61 E-04	5.67 E-04	MS, RI

1-Hepten-6-one, 2-methyl-	10408-15-8	C8H14O	Ketones	648.621	Fruity, fresh, citrusy	Fruity, Fresh	957	966	1.29	4.61 E-05	1.85 E-04	MS, RI
2-Hexenal	505-57-7	C6H10O	Aldehydes	359.283	Green, grassy, and fruity	Green, Fruity	843	847-854	1.29	5.03 E-04	1.48 E-03	MS, RI
1-Hexanol	111-27-3	C6H14O	Alcohols	399.52	Fruity, floral, and slightly waxy	Fruity, Floral	861	865	1.28	2.71 E-06	1.50 E-05	MS, RI, Std
5-Heptenal, 2,6-dimethyl-	106-72-9	C9H16O	Aldehydes	905.897	Melon scent	Fruity	1045	1053-1060	1.27	9.23 E-07	5.94 E-06	MS, RI
3-Hexen-1-ol, benzoate, (Z)-	25152-85-6	C13H16O2	Esters	2300.06	Fresh, green, grassy, slightly fruity	Fresh, Green, Fruity	1562	1571	1.26	4.21 E-04	1.25 E-03	MS, RI
Benzaldehyde, 2,4,6-trimethyl-	487-68-3	C10H12O	Aldehydes	1656.42	Sweet, almond-like	Sweet, Nutty	1305	1323	1.25	3.49 E-06	1.83 E-05	MS, RI
Butanoic acid, 2-methyl-, hexyl ester	10032-15-2	C11H22O2	Esters	1443.09	Fruity and sweet	Fruity, Sweet	1228	1235	1.22	4.20 E-06	2.13 E-05	MS, RI
Heptanal	111-71-7	C7H14O	Aldehydes	471.293	Fruity, pungent, and slightly fatty	Fruity	894	894-914/902-904	1.19	2.83 E-04	8.83 E-04	MS, RI, Std
2(3H)-Furanone, 5-ethyldihydro-	695-06-7	C6H10O2	Heterocyclic compounds	901.502	Sweet, caramel-like, fruity	Sweet, Fruity	1044	1047-1068	1.18	4.52 E-07	3.18 E-06	MS, RI
(E)-β-Famesene	18794-84-8	C15H24	Terpenoids	2013.78	Woody	Woody	1443	1457-1471	1.18	2.35 E-04	7.65 E-04	MS, RI
Ethanone, 1-(2-hydroxy-5-methoxyphenyl)-	705-15-7	C9H10O3	Ketones	1991.99	Unknown	unknown	1435	1422	1.17	1.80 E-03	4.49 E-03	MS, RI

2-Pentenal, (E)-	1576-87-0	C5H8O	Aldehydes	204.354	Fruity, green, and floral	Fruity, Green, Floral	751	759	1.16	7.02 E-04	1.92 E-03	MS, RI, Std
3-Penten-2-one, 4-methyl-	141-79-7	C6H10O	Ketones	255.029	Sweet, floral, and fruity	Sweet, Floral, Fruity	771	792-800	1.16	2.05 E-04	6.90 E-04	MS, Std
β-Ocimene	13877-91-3	C10H16	Terpenoids	886.679	Fruity, citrusy, woody	Fruity, Woody	1039	1023-1050	1.14	8.11 E-05	3.10 E-04	MS, RI, Std
2-Undecenal, E-	53448-07-0	C11H20O	Aldehydes	1791.65	pungent, fatty, and somewhat citrus-like	Fruity	1357	1361-1366	1.13	6.29 E-04	1.78 E-03	MS, RI
2,4-Heptadienal, (E,E)-	4313-03-5	C7H10O	Aldehydes	739.27	Fatty, flowery	Floral	989	992-1019	1.11	2.11 E-03	5.22 E-03	MS, RI
1-Undecene, 4-methyl-	74630-39-0	C12H24	Alkanes	1042.83	Unknown	unknown	1091	1085	1.10	2.60 E-04	8.29 E-04	MS, RI
2,4-Heptadienal, (E,E)-	4313-03-5	C7H10O	Aldehydes	784.124	Fatty, flowery	Floral	1005	983-1019	1.10	1.32 E-03	3.45 E-03	MS, RI
1,2-Benzenedicarboxylic acid, bis(2-methylpropyl) ester	84-69-5	C16H22O4	Esters	2914.79	mild, slightly floral and fruity	Floral, Fruity	1845	1869	1.08	1.01 E-02	2.01 E-02	MS, RI
Oxalic acid, allyl nonyl ester	0	C14H24O4	Esters	1821.51	Unknown	unknown	1368	1376-1382	1.08	1.28 E-03	3.38 E-03	MS, RI
3-Tetradecen-5-yne, (E)-	22822-99-7	C11H18	Olefins	3369.24	Unknown	unknown	2082	2098	1.07	1.51 E-04	5.41 E-04	MS, RI

Analyte 1410	96-76-4	C14H22O	Phenols	2159.83	Sharp antiseptic	Pungent	1502	1502-1517	1.06	1.89 E-04	6.47 E-04	MS, RI
Hexanoic acid, 3-hexenyl ester, (Z)-	31501-11-8	C12H22O2	Esters	1831.47	Fruity, green	Fruity, Green	1372	1379-1386	1.04	2.31 E-04	7.60 E-04	MS, RI, Std
Nerolidol	7212-44-4	C15H26O	Terpenoids	2346.43	Pleasant, floral	Floral	1582	1562-1573	1.00	2.41 E-03	5.80 E-03	MS, RI

Supplementary Table S3. List of sensory differentiating metabolites for light-scented Tieguanyin tea between high-quality (tope 30 in aroma score) and low quality (bottom 30 in aroma score).

Compounds	CAS No.	Formula	Metabolite Class	RT	Odor description	Category	RIexp	RIlit	Vip	p. value	FDR	Identified Method
trans-3-Hexenyl butyrate	16491-36-4	C10H18O2	Esters	1299.49	Fresh clean	Fresh	1178	1179-1189	2.4	6.66 E-07	6.86 E-05	MS, RI, Std
(E)-β-Famesene	18794-84-8	C15H24	Terpenoids	2013.78	Woody	Woody	1443	1457-1471	2.3	1.08 E-06	8.33 E-05	MS, RI
trans-2-Hexenyl hexanoate	53398-86-0	C12H22O2	Esters	1853.88	Green	Green	1380	1391	2.3	1.98 E-07	3.05 E-05	MS, RI, Std
Hexyl hexanoate	6378-65-0	C12H24O2	Esters	1846.09	Fresh cut grass	Fresh, Green	1377	1384-1387	2.0	4.29 E-04	4.90 E-03	MS, RI, Std
Hexyl 2-methyl butyrate	10032-15-2	C11H22O2	Esters	1443.09	Fruity, sweet, and apple-like	Fruity, Sweet	1228	1235	2.0	4.43 E-05	9.13 E-04	MS, RI
Nerolidol	7212-44-4	C15H26O	Terpenoids	2346.43	Pleasant, floral	Floral	1582	1562-1573	1.9	1.27 E-05	4.90 E-04	MS, RI
cis-α-Bisabolene	25532-79-0	C15H24	Terpenoids	2223.41	maybe pleasant, citrus-like	Fruity	1530	1512	1.9	3.78 E-04	4.90 E-03	MS, RI

cis-3-Hexenyl cis-3-hexenoate	61444-38-0	C12H20O2	Esters	1840.46	Green, fruity, and slightly floral	Green, Fruity, Floral	1375	1388	1.8	9.45 E-04	9.39 E-03	MS, RI
3,5-Octadien-2-one	38284-27-4	C8H12O	Ketones	1032.35	Fruity, green, grassy, sweet	Fruity, Green, Sweet	1087	1068-1073	1.8	1.90 E-05	6.52 E-04	MS, RI
Neophytadiene	504-96-1	C20H38	Terpenoids	2873.65	Mild, woody, slightly citrusy	Woddy, Fruity	1825	1836-1838	1.8	4.29 E-04	4.90 E-03	MS, RI
2H-Pyran-2-one, tetrahydro-6-(2-pentenyl)-, (Z)-	25524-95-2	C7H14O	Heterocyclic compounds	2110.4	Fruity, sweet, floral	Fruity, Sweet, Floral	1482	1494-1517	1.7	1.60 E-07	3.05 E-05	MS, RI
Analyte 1233	469-61-4	C15H24	Terpenoids	1911.4	NA	NA	1402	1409	1.7	1.13 E-03	9.94 E-03	MS, RI
2-Undecenal, E-	53448-07-0	C11H20O	Aldehydes	1791.65	citrusy, orange-like scent, fresh	Fruity, Fresh	1357	1361-1366	1.7	6.17 E-03	3.47 E-02	MS, RI
(R,S)-5-Ethyl-6-methyl-3E-hepten-2-one	57283-79-1	C10H18O	ketones	1170.08	Fruity, green, and slightly floral	Fruity, Green,Floral	1134	1146	1.7	6.47 E-03	3.51 E-02	MS, RI
Oxalic acid, allyl nonyl ester	0	C14H24O4	Esters	1821.51	unknown, maybe sweet, fruity	Fruity	1368	1376-1382	1.7	2.59 E-03	1.86 E-02	MS, RI

Furan, 2-pentyl-	3777-69-3	C9H14O	Heterocyclic compounds	717.024	citrus	Fruity	981	972-996	1.6	5.07E-03	3.01E-02	MS, RI
α-Ionone	127-41-3	C13H20O	Terpenoids	1934.01	Flowery, violet-like	Floral	1411	1418-1456	1.6	3.04E-03	2.09E-02	MS, RI
Undecane, 2-methyl-	7045-71-8	C12H26	Alkanes	1240.53	Little to no odor	No odor	1158	1163-1165	1.6	7.84E-03	4.11E-02	MS, RI
2H-Pyran-2-one, tetrahydro-6-pentyl-	705-86-2	C10H18O2	Heterocyclic compounds	2114.52	Caramel-like, toasty, nutty	Nutty	1484	1463-1510	1.5	3.74E-03	2.41E-02	MS, RI
β-Ionone	79-77-6	C13H20O	Terpenoids	2076.09	Flowery, violet-like	Floral	1469	1469-1512	1.5	1.77E-03	1.34E-02	MS, RI
Tetradecane	629-59-4	C16H34	Alkanes	1886.25	Little to no odor	Little odor	1393	1400	1.5	3.53E-05	8.40E-04	MS, RI
3,5-Octadien-2-one, (E,E)-	30086-02-3	C8H12O	Ketones	957.797	Fruity, green, grassy	Fruity, Green	1062	1068-1073	1.5	1.00E-03	9.39E-03	MS, RI
1,3,6,10-Dodecatetraene, 3,7,11-trimethyl-, (Z,E)-	26560-14-5	C15H24	Terpenoids	2108.98	Little to no odor	Little odor	1482	1462-1491	1.5	1.60E-04	2.48E-03	MS, RI

Tridecanoic acid, methyl ester	1731-88-0	C14H28O2	Esters	3050.93	Oily, waxy, fatty	Unpleasant	1913	1924-1926	1.4	3.32 E-04	4.67 E-03	MS, RI
Isophytol	505-32-8	C20H40O	Terpenoids	3090.54	Mild, waxy, odorless	Little odor	1934	1938-1950	1.4	5.07 E-03	3.01 E-02	MS, RI
2(3H)-Furanone, 5-ethenyldihydro-5-methyl-	1073-11-6	C7H10O2	Heterocyclic compounds	853.877	Sweet, fruity, and caramel-like	Fruity, Sweet	1028	1046-1071	1.4	5.17 E-04	5.51 E-03	MS, RI
Indole	120-72-9	C8H7N	Heterocyclic compounds	1607.44	Light jasmine	Floral	1287	1292	1.4	4.36 E-03	2.69 E-02	MS, RI, Std
Tridecane, 3-methyl-	6418-41-3	C10H14O	Alkanes	2044.34	Little to no odor	Little odor	1456	1371-1375	1.3	1.34 E-03	1.12 E-02	MS, RI
beta-Nerolidol	40716-66-3	C15H26O	Alcohols	2291.55	Floral, herbal, slightly woody	Floral	1559	1562	1.3	6.17 E-03	3.47 E-02	MS, RI
2-Nonenal, (E)-	60784-31-8	C9H16O	Aldehydes	1226.24	Strong, unpleasant, and rancid	Unpleasant	1153	1151	1.1	3.11 E-04	4.58 E-03	MS, RI
cis-3-Hexenyl isovalerate	53398-85-9	C11H20O2	Esters	1427.59	Green, fruity and reminiscent	Gree, Fruity	1223	1233	1.1	4.78 E-05	9.22 E-04	MS, RI

					of apple or pear							

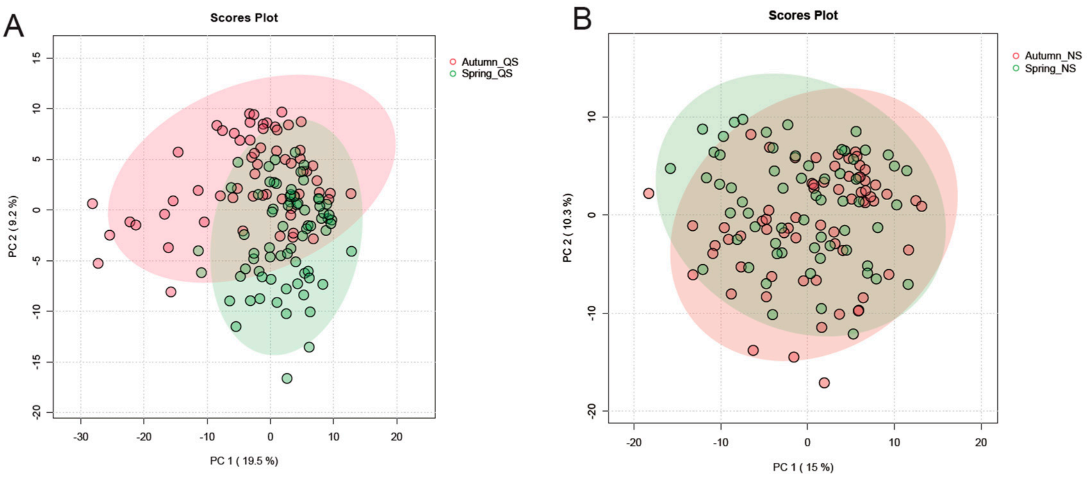

Figure S1. PCA plot for tieguanyin oolong tea made from leaves harvested in spring and autumn. A: light scented tea, B: strong scented tea.

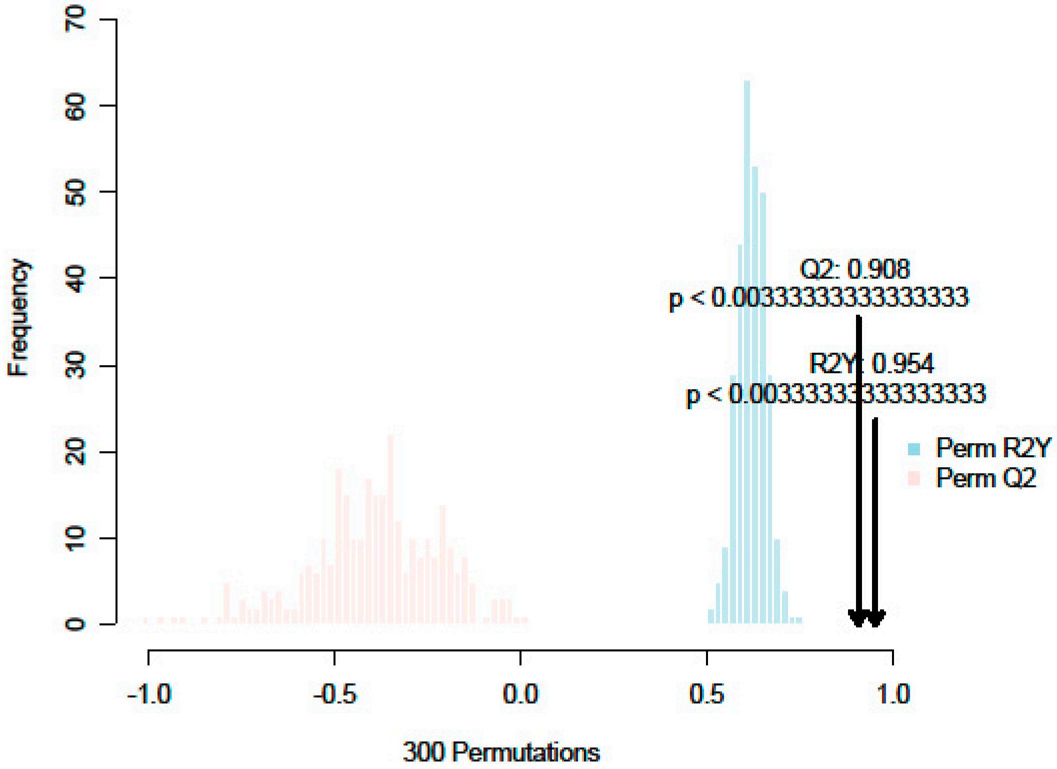

Figure S2. premutation test of OPLSDA for light scented tea.

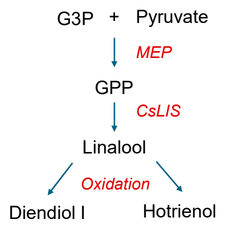

Figure S3. A scheme of transformation between linalool related products.
